# Recovery of sinus rhythm by tafamidis in patients with wild-type transthyretin amyloid cardiomyopathy with atrial arrhythmias

**DOI:** 10.1093/omcr/omac007

**Published:** 2022-02-19

**Authors:** Yoshitaka Isotani, Eisuke Amiya, Junichi Ishida, Masato Ishizuka, Masaru Hatano, Koki Nakanishi, Masao Daimon, Issei Komuro

**Affiliations:** Department of Cardiovascular Medicine, Graduate School of Medicine, University of Tokyo, , Hongo 7-3-1, Bunkyo-ku, Tokyo, Japan; Department of Cardiovascular Medicine, Graduate School of Medicine, University of Tokyo, , Hongo 7-3-1, Bunkyo-ku, Tokyo, Japan; Department of Therapeutic Strategy for Heart Failure, University of Tokyo, Hongo 7-3-1, Bunkyo-ku, Tokyo, Japan; Department of Cardiovascular Medicine, Graduate School of Medicine, University of Tokyo, , Hongo 7-3-1, Bunkyo-ku, Tokyo, Japan; Department of Cardiovascular Medicine, Graduate School of Medicine, University of Tokyo, , Hongo 7-3-1, Bunkyo-ku, Tokyo, Japan; Department of Cardiovascular Medicine, Graduate School of Medicine, University of Tokyo, , Hongo 7-3-1, Bunkyo-ku, Tokyo, Japan; Advanced Medical Center for Heart Failure, University of Tokyo, Hongo 7-3-1, Bunkyo-ku, Tokyo, Japan; Department of Cardiovascular Medicine, Graduate School of Medicine, University of Tokyo, , Hongo 7-3-1, Bunkyo-ku, Tokyo, Japan; Department of Cardiovascular Medicine, Graduate School of Medicine, University of Tokyo, , Hongo 7-3-1, Bunkyo-ku, Tokyo, Japan; Department of Clinical Laboratory Medicine, University of Tokyo, Hongo 7-3-1, Bunkyo-ku, Tokyo, Japan; Department of Cardiovascular Medicine, Graduate School of Medicine, University of Tokyo, , Hongo 7-3-1, Bunkyo-ku, Tokyo, Japan

## Abstract

Transthyretin amyloid cardiomyopathy (ATTR-CM) is a life-threatening infiltrative disease in elderly patients. Atrial arrhythmias (AAr) are common in patients with ATTR-CM. However, AAr treatment in these patients is challenging. In this case, a patient diagnosed with wild-type ATTR-CM suffered atrial fibrillation (AF) for ~1 year, according to the data of his self-monitoring and regular electrocardiogram. This AF reverted to normal sinus rhythm a few months after the initiation of tafamidis without administering an antiarrhythmic drug. Tafamidis may be beneficial as alternative antiarrhythmic therapy in patients with ATTR-CM.

## INTRODUCTION

Atrial arrhythmias (AAr), which include atrial fibrillation (AF), atrial flutter and atrial tachycardia, are common in patients diagnosed with cardiac amyloidosis, especially those with wild-type transthyretin amyloid cardiomyopathy (ATTR-CM) [1]. The maintenance of sinus rhythm (SR) is associated with improved survival among patients with AAr [2]. However, rhythm control therapy for AAr in ATTR-CM is challenging.

## CASE REPORT

In 2018, a 70-year-old man with a history of hypertension presented with leg edema. Echocardiography revealed left ventricular (LV) hypertrophy. The patient’s medications, including diuretics, were adjusted accordingly.

**Figure 1:**
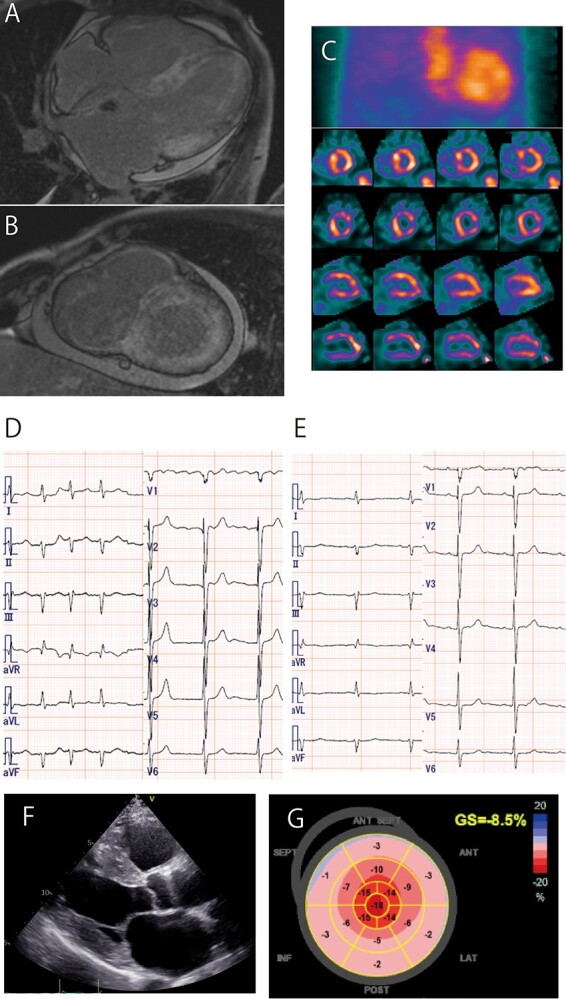
(A, B) Cardiac magnetic resonance imaging showing late gadolinium enhancement in the left ventricle, right ventricle and atrial walls. (C) Technetium hydroxymethylene diphosphonate showing diffuse radiotracer uptake in the myocardium. (D) Electrocardiography showing atrial fibrillation. (E) Recovery of sinus rhythm 3 months after the initiation of tafamidis. (F) Echocardiography showing left ventricular hypertrophy and left atrium dilatation. (G) Relative apical sparing by longitudinal speckle tracking strain imaging.

In 2019, his leg edema worsened. Coronary angiography did not reveal significant stenosis. Cardiac magnetic resonance (CMR) identified late gadolinium enhancement (LGE) with diffuse subendocardial enhancement in the walls of the left and right ventricles and atria (Fig. 1A and B). Technetium-99 hydroxymethylene diphosphonate imaging demonstrated diffuse, strong uptake in the myocardium (Fig. 1C). An endomyocardial biopsy confirmed amyloid deposits, and a gene test revealed wild-type ATTR-CM.

In March 2020, electrocardiography revealed AF for the first time (Fig. 1D). Laboratory data showed that the level of thyroid stimulating hormone was mildly elevated (5.17 μIU/ml), whereas free triiodothyonine and thyroxine were within normal range. LV systolic dysfunction with an ejection fraction (EF) of 32%, concentric LV hypertrophy, and left atrium dilatation with a relative apical sparing pattern with global longitudinal strain was found with echocardiography (Fig. 1F and G). The patient checked his heart rate every day, which verified AF was continuous without recovering SR. In November 2020 (9 months after the development of AF), 80 mg tafamidis was initiated with no adverse effects.

Three months after starting tafamidis, his heart rate, measured by self-monitoring, demonstrated a sudden decrease to 50 bpm. Electrocardiography showed that AF reverted to normal SR (the total duration of AF was about 1 year) without antiarrhythmic drugs and beta-blocker (Fig. 1E). Normal SR has been maintained for > 6 months. However, left atrium dilatation did not significantly change. A follow-up right heart catheterization demonstrated increased mean right atrial pressure (17 mm Hg) and mean pulmonary artery wedge pressure (28 mm Hg). These increased levels were comparable with those measured before tafamidis treatment. Echocardiographic parameters, including LV global longitudinal strain and diastolic function, magnetic resonance imaging (MRI) findings, and biomarkers such as BNP and troponin I, did not change (Table 1). A sodium–glucose cotransporter-2 inhibitor was administered for water retention.

**Table 1:** Parameters before and after the initiation of tafamidis

Parameter	Before the initiation of tafamidis	After the initiation of tafamidis
	AF rhythm	Sinus rhythm
Echocardiography		
LVDd (mm)	42	43
LVDs (mm)	35	37
LVEF (Biplane)	32%	39%
IVST (mm)	14	14
PWT (mm)	13	14
LAD (mm)	47	48
LAVI (ml/m^2^)	58	61
MR grade	Trivial	Trivial
LV-GLS	−8.50%	−7.60%
Biomarker		
BNP (pg/ml)	168.2	284.6
Troponin I (pg/ml)	144.0	125.6
Right heart catheterization		
mean RAP (mm Hg)	16	17
mean PAWP (mm Hg)	29	28
mean PAP (mm Hg)	37	37
CO (Fick) (L/min)	3.33	3.49
CI (Fick) (L/min/m^2^)	1.87	1.97
MRI		
LVEF	42.38%	46.50%
LVEDV (ml)	193.5	169.7
LVESV (ml)	111.5	90.8
LV mass (g)	185.6	176.4

Dd, end-diastolic diameter; Ds, end-systolic diameter; EF, ejection fraction; IVST, interventricular septum thickness; PWT, posterior wall thickness; LAD, left atrium diameter; LAVI, left atrium volume index; MR, mitral regurgitation; GLS, global longitudinal strain; RAP, right atrium pressure; PAWP, pulmonary artery wedge pressure; PAP, pulmonary artery pressure; CO, cardiac output; CI, cardiac index; EDV, end-diastolic volume; ESV, end-systolic volume.

## DISCUSSION

Wild-type ATTR-CM has a high prevalence of AAr, including AF, which is extremely high, about 70–90% [3]. Donnellan *et al.* suggested AF as a sign of ATTR-CM advancement. Ablation therapy was reported to be effective, particularly in the early phase, suggesting that some interventions can revert this complication [4]. However, the recurrence rate is extremely high; therefore, strategies for this complication are challenging [5].

In the Transthyretin Amyloidosis Cardiomyopathy Clinical Trial (ATTR-ACT), tafamidis significantly reduced mortality and cardiovascular-related rehospitalizations in ATTR-CM patients [6]. Further analysis showed a reduction in hospitalizations due to arrhythmias [7]. The percentage of patients whose SR was maintained was higher in those who took tafamidis after ablation therapy than those who did not [2]. This fact supports the use of tafamidis in AAr therapy of ATTR-CM.

In addition to the current case, we experienced another case of a patient with ATTR-CM complicating with atrial arrythmia. Eighty-year-old man with ATTR-CM demonstrated the recovery of SR spontaneously two months after tafamidis treatment, whose atrial flutter lasted about 1 year.

Our two cases share similarities. First, the time from detection of AAr to tafamidis administration was almost the same, i.e. <1 year. Second, SR was restored a few months after the initiation of tafamidis. Two AF mechanisms in cardiac amyloidosis patients have been proposed, increased LV filling pressure leading to atrial wall dilatation and direct amyloid influences in the left atrium that disrupts myocardial conduction. Both mechanisms may make AF treatment in cardiac amyloidosis more difficult. In this case, there were few findings regarding the improvement of heart failure by tafamidis. Therefore, the recovery of SR did not derive from the improvement of heart failure. The effect of tafamidis in these cases was considered to be directly related to atrial electrical arrhythmogenicity.

Recent reports suggested amyloid organ deposition consists of a multistep process. It is also known that organ damage occurs at the stage of nonfibrillar TTR before the amyloid deposition in the organ. Nonfibrillar TTR seems to play an important role in enhancing the leakage of circulating TTR into extracellular spaces by affecting endothelial cells of blood vessels [8]. This means that tafamidis may reduce organ damage relatively early in the course of treatment by reducing the amount of nonfibrillar TTR. Indeed, Rettl R suggested that the treatment with tafamidis for a period of only 6 months may have positive effects on the value of extra cellular volume fraction of MRI, which might suggest the regression of TTR deposition [9]. This mechanism could be the reason for the restoration of SR in our cases, which should be verified by more robust research.

The certifying the impact of a specific therapy on the recovery of SR is generally difficult. There had been little reports about the spontaneous conversion of long-standing AF, which described it as very uncommon phenomenon [10]. Furthermore, atrial amyloid deposition in ATTR-CM might make the recovery of SR more difficult as compared with patients other than ATTR-CM [2]. Indeed, Krishnappa demonstrated all cases of AF with amyloid deposition corresponded to persistent AF and the progression of ATTR-CM corresponded to the development of AF [11]. In the current case, the hemodynamic parameters did not change before and after the administration of tafamidis, which indicated that the recovery of SR was not attributable to hemodynamic changes. As a result, the effect of tafamidis on the recovery of SR might be one possible mechanism to be considered.

Of course, there is a limitation that the verification of AF persistence is generally difficult. The possibility that it was actually paroxymal AF cannot be completely denied, which, however, is considered to be unlikely.

In conclusion, tafamidis may be an effective drug to regain SR. Further research is necessary to support this.

## DISCLOSURES

EA belongs to the Department, endowed by NIPRO-Corp, Terumo-Corp., Senko-Medical-Instrument-Mfg., Century-Medical, Inc., ONO-pharmaceutical-Co., Ltd Medtronic-JAPAN Co., Ltd, Nippon-Shinyaku Co., Ltd, Abiomed-Inc, AQuA-Inc, Fukuda-Denshi Co., Ltd, and Sun-Medical-Technology-Research Corp. EA got lecture fee from Pfizer Inc. JI got lecture fee and research grant (55026059) from Pfizer Inc.

## CONSENT

Written consent obtained.

## GUARANTOR

Guarantor is Eisuke Amiya.
